# In Vitro Cell Culture Model for Osteoclast Activation during Estrogen Withdrawal

**DOI:** 10.3390/ijms25116134

**Published:** 2024-06-01

**Authors:** Nisha Gandhi, Safia Omer, Rene E. Harrison

**Affiliations:** 1 Department of Cell & Systems Biology, University of Toronto Scarborough, Toronto, ON M1C 1A4, Canada; nisha.gandhi@mail.utoronto.ca; 2 Department of Biological Sciences, University of Toronto Scarborough, Toronto, ON M1C 1A4, Canada; safia.omer@utoronto.ca

**Keywords:** osteoclast, estrogen, osteoporosis, cytoskeleton

## Abstract

Estrogen (17β-estradiol) deficiency post-menopause alters bone homeostasis whereby bone resorption by osteoclasts exceeds bone formation by osteoblasts, leading to osteoporosis in females. We established an in vitro model to examine the consequences of estrogen withdrawal (E2-WD) on osteoclasts derived from the mouse macrophage RAW 264.7 cell line and utilized it to investigate the mechanism behind the enhanced osteoclast activity post-menopause. We found that a greater population of osteoclasts that underwent E2-WD contained a podosome belt necessary for osteoclasts to adhere and resorb bone and possessed elevated resorptive activity compared to osteoclasts exposed to estrogen (E2) continuously. Our results show that compared to osteoclasts that received E2 continuously, those that underwent E2-WD had a faster rate of microtubule (MT) growth, reduced RhoA activation, and shorter podosome lifespan. Thus, altered podosome and MT dynamics induced by the withdrawal of estrogen supports podosome belt assembly/stability in osteoclasts, which may explain their enhanced bone resorption activity.

## 1. Introduction

The skeleton serves a variety of functions including structural support, protection of vital internal organs, enablement of movement, and regulation of mineral homeostasis within the body [1]. Although inert in appearance, bone is a highly dynamic organ that undergoes remodeling through the orchestrated activities of osteoclasts; the specialized bone-resorbing cells and osteoblasts; the bone-forming cells. Mature osteoclasts resorb the bone matrix via the secretion of acid and proteolytic enzymes, forming resorption pits [2]. Deregulated osteoclast activity results in osteoporosis, a common bone disease that is characterized by low bone mass, altered microarchitecture of bone tissue, and increased bone fragility [3,4]. At a cellular level, osteoporosis results from osteoclastic bone resorption uncompensated for by osteoblastic bone formation [4]. The shift in the dynamic equilibrium of bone formation and resorption can be attributed to several factors, notably estrogen deficiency post-menopause [5].

Estrogen is produced primarily by the ovaries in addition to non-gonadal organs such as the liver, brain, and bone [6,7]. 17β-estradiol (E2), the most prevalent form of estrogen in premenopausal women, is produced by the ovaries and released to target distal estrogen-responsive tissues including reproductive and non-reproductive organs. Alternatively, extra-gonadal-synthesized E2 acts locally to regulate tissue-specific functions [6]. Interestingly, human and rat osteoblastic cell lines express the estrogen synthetase aromatase that mediates the conversion of androgens to E2 [8,9,10], suggesting localized E2 synthesis in bone cells.

To activate signaling pathways, E2 binds to the nuclear estrogen receptors (ER) alpha and beta (ERα and ERβ) in the cytoplasm and induces the formation of a homo/heterodimer complex of two ERs [11], which translocate to the nucleus to bind at estrogen response elements (EREs) within target genes [12,13]. In addition to the genomic estrogen-mediated signaling, E2 exerts rapid effects, within seconds or minutes, through nongenomic signaling. This occurs via plasma membrane-associated ERs [14] or G-protein-coupled estrogen receptor (GPER1) [15,16] that induces nongenomic effects through intracellular PI3K/AKT and MAPK signaling, stimulating downstream processes such as cell death, protein synthesis, calcium release, or the activation of calcium-calmodulin-dependent kinases [14].

E2 is a positive regulator of bone homeostasis, as it slows the rate of bone resorption and maintains the balance between bone resorption and bone formation [17]. E2 has a pro-apoptotic effect on osteoclasts, and osteoclast-specific deletion of ERα in mice results in an increase in the number of osteoclasts and bone mass reduction [18,19]. Mechanistically, E2 stimulates the production of transforming growth factor-beta1, which promotes the apoptosis of osteoclasts [20,21,22]. E2 also downregulates the formation of osteoclasts by directly suppressing nuclear factor kappa-Β ligand (RANKL)-induced osteoclast differentiation [23,24,25], suppressing osteoclast adhesion to the bone matrix [26], and reducing bone resorption by disrupting the formation of actin-rich sealing zones and podosome density [27]. The mechanism by which E2 disrupts adhesion and sealing zone formation is unclear; however, a recent study suggested that E2 inhibits c-Src/Vav3/Rac1 signaling in osteoclasts [28], which is necessary for cytoskeletal organization. Given the significant influence of E2 on bone cells, the marked decline in E2 production by the ovaries in post-menopausal females is recognized as the major cause of post-menopausal osteoporosis.

The impacts of E2 loss on bone cell formation and function has largely been investigated using ovariectomized mice models or primary cell lines exposed acutely to estrogen [18,20,21,29,30]. Estrogen deficiency in vitro can be achieved by pre-treatment of cells with E2 followed by estrogen withdrawal (E2-WD). Multiple in vitro E2-WD models in osteoblast and osteocyte cell cultures have been established to study estrogen loss effects on these cell types [31,32]. For our study, our first aim was to develop a E2-WD system in vitro using cultured cells to understand both how the presence of estrogen suppresses osteoclasts and to mimic and understand osteoclast activation after withdrawal (simulating post-menopause). We used the mouse macrophage RAW264.7 cell line, which robustly differentiates into multinucleated osteoclasts upon receptor activator of RANKL stimulation within 4 days of plating [33]. After establishing a cell culture model where osteoclast activity was suppressed by E2 and subsequently enhanced upon withdrawal, we investigated the cellular mechanism(s) behind these estrogen-altering phenotypes.

## 2. Results

### 2.1. Continuous Estrogen (E2) Treatment during Differentiation Reduced Multinucleated Osteoclast Formation and Resorption That Was Reversed Following E2 Withdrawal (E2-WD)

**Figure 1:**
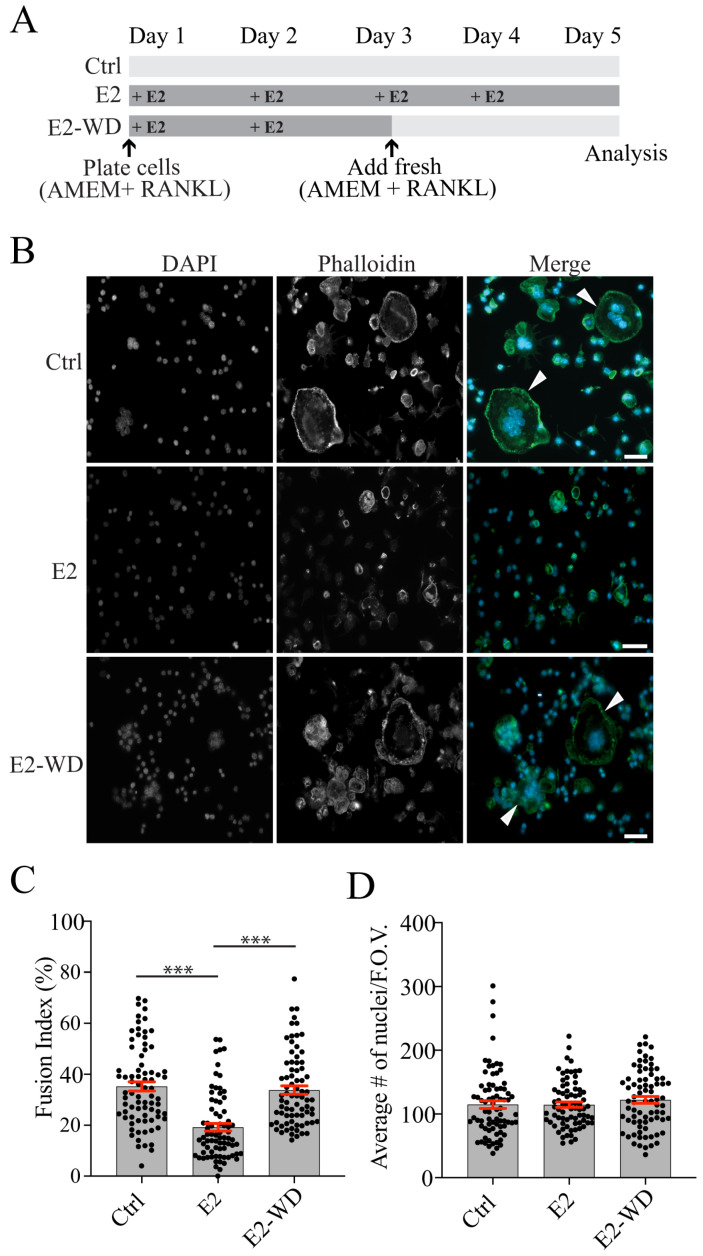
Osteoclasts undergoing estrogen withdrawal (E2-WD) had a higher fusion index than did osteoclasts continuously treated with 17β-estradiol (E2). (**A**) Schematic of the in vitro protocol used to generate the E2-WD model. On Day 1, RAW 264.7 cells were plated on 12-well plates in AMEM containing 25 ng/mL of RANKL only (Ctrl) and together with 10 nM of 17β-estradiol on Days 1–5 (E2) and on Days 1–2 (E2-WD). On Day 3, the medium was replenished with RANKL (Ctrl and E2-WD) or RANKL and 10 nM of 17β-estradiol (E2). Experiments were terminated on day 5. (**B**) Representative immunofluorescence images of untreated osteoclasts (Ctrl), osteoclasts continuously treated with 17β-estradiol (E2), and osteoclasts that underwent estrogen withdrawal (E2-WD). Cells were fixed after a 4-day differentiation period and stained with DAPI (nuclei) and phalloidin (F-actin). Arrowheads indicate large osteoclasts. (**C**) Quantification of the fusion index (number of nuclei in osteoclasts (≥3 nuclei) divided by the total number of nuclei per field of view (F.O.V.) for each condition. (**D**) Graph showing the average number of nuclei per F.O.V. for each treatment. Data represent the mean ± SEM from three biological replicates. *p*-values were calculated using the one-way ANOVA test followed by Tukey’s multiple comparison test (*n* ≥ 75, *** *p* < 0.0001). Scale bars = 50 μm.

To understand the mechanisms driving osteoclast activation in osteoporosis, we developed an in vitro estrogen withdrawal (E2-WD) cellular model. In vitro E2-WD models using MLO-Y4 and MC3T3-E1 cell lines have been established to examine estrogen loss effects in osteocytes and osteoblasts, respectively [32,34]. To study osteoclasts, we utilized the immortalized murine RAW 264.7 (RAW) macrophage cell line, which we and others have shown to respond to RANKL stimulation in vitro to generate functional osteoclasts [35,36,37]. In our E2-WD model (Figure 1A), RAW cells were stimulated with RANKL and treated with 10 nM of 17β-estradiol (E2), a concentration within the physiological range in mouse serum [38] for 2 days (Days 1 and 2), which was followed by withdrawal (ED-WD) for the subsequent 2 days (Days 3 and 4). On Day 5, cells were fixed and stained for DAPI and F-actin (i.e., phalloidin) (Figure 1B). There were fewer multinucleated osteoclasts in cells continuously treated with E2 compared to those in the control group (Ctrl) that constituted fully activated osteoclasts (Figure 1B). There was also a marked reduction in the number of nuclei in osteoclasts under continuous E2 treatment compared to that in the Ctrl group (Figure 1B). Osteoclasts that underwent E2-WD had a greater number of multinucleated osteoclasts than did those under continuous E2 treatment (Figure 1B). The fusion index (%) was determined at Day 5 for each treatment condition, and cells with three or more nuclei were considered osteoclasts (Figure 1C). The fusion index was significantly reduced (~55%) in cells continuously treated with E2 compared to Ctrl cells (Figure 1C). In cells under E2-WD, there was a significant increase in fusion compared to the E2 group (Figure 1C). There was no impact of continuous E2 treatment or E2-WD on cell proliferation/viability, as the average number of nuclei per field of view (F.O.V.) was similar among all three groups (Figure 1D).

**Figure 2:**
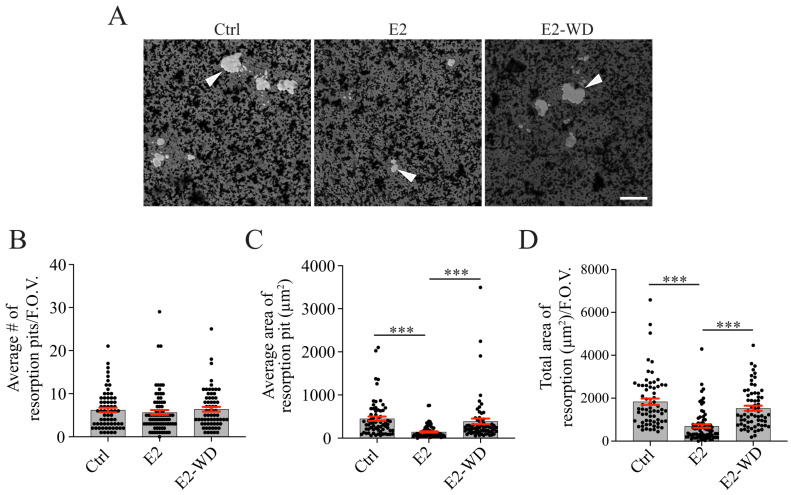
Osteoclasts under E2-WD exhibited enhanced mineral resorption compared to resorption by osteoclasts continuously treated with E2. (**A**) Representative brightfield images of resorption pits formed by the osteoclasts for each condition. Arrowheads indicate regions of resorption. (**B**–**D**) Graphs displaying the average number of resorption pits (*n* ≥ 65) per F.O.V., average area of each resorption pit (μm^2^), and total area of resorption (μm^2^) per F.O.V. Data are presented as the mean ± SEM from three biological replicates. *p*-values were calculated using the one-way ANOVA test followed by Tukey’s multiple comparison test (*** *p* < 0.0001). Scale bar = 100 μm.

We next assayed our model for the impacts of E2 withdrawal on osteoclast activation, namely bone resorption. We examined this by differentiating RAW cells on calcium phosphate (CaP)-coated coverslips in the presence of RANKL and with our established E2 treatments. On Day 5, osteoclasts were lifted, and CaP-coated coverslips were stained with silver nitrate to observe areas resorbed using brightfield imaging (Figure 2A). The average number of discrete resorption pits per F.O.V. were not significantly different between the three conditions (Figure 2B). However, osteoclasts under continuous E2 treatment had a significantly smaller average area of resorption pits (Figure 2C) and resorbed a smaller total area of bone mimetic per F.O.V. compared to the Ctrl osteoclasts (Figure 2D). Interestingly, osteoclasts that underwent E2-WD had a significantly larger average area of resorption pits (Figure 2C) and resorbed a larger total area of bone mimetic per F.O.V. (Figure 2D) in comparison to the E2 group. The average area of resorption pits (µm^2^) in fully activated Ctrl cells versus cells under E2-WD were not significantly different (Figure 2C). Similarly, there was no significant difference in the total area resorbed per F.O.V. by fully activated Ctrl cells compared to cells under E2-WD (Figure 2D).

### 2.2. The Effects of Estrogen on Osteoclast-Specific Gene Expression In Vitro

**Figure 3:**
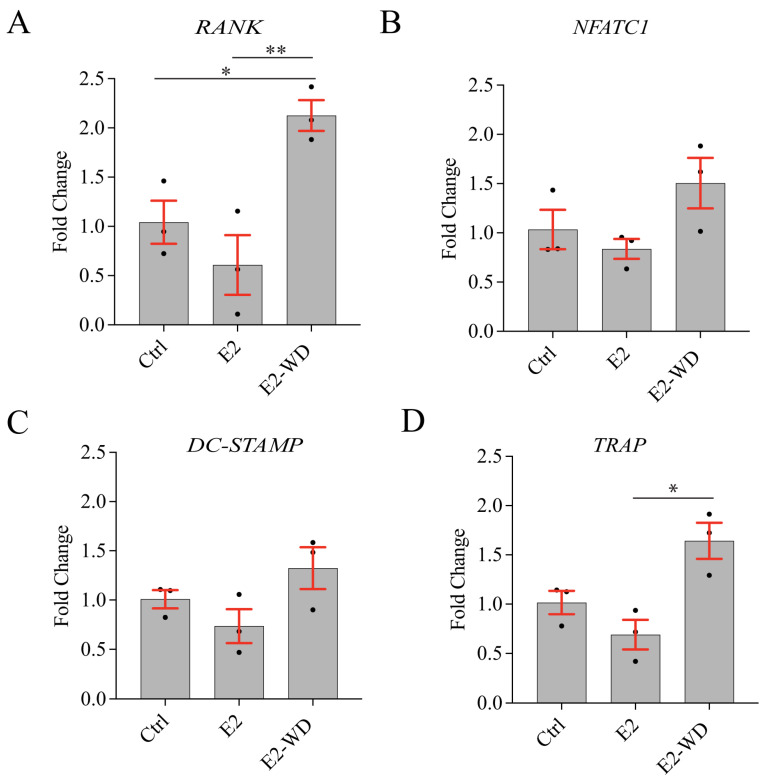
Increased gene expression of RANK and TRAP in multinucleated osteoclasts under E2-WD compared to multinucleated osteoclasts continuously treated with E2. (**A**–**D**) qRT-PCR analysis of RANK, NFATC1, DC-STAMP, and TRAP was performed on Day 5 imultinucleated osteoclasts in each experimental condition. Expression levels were normalized using GAPDH, and the fold-change was calculated relative to the Ctrl condition. Data represent the mean ± SEM from three independent replicates. *p*-values were calculated using the one-way ANOVA test followed by Tukey’s multiple comparison test (* *p* < 0.05 and ** *p* < 0.001).

Next, we examined whether the upregulated osteoclast activity under E2-WD, compared to E2-treated cells could be explained by alterations in osteoclast-specific gene expression. qRT-PCR was performed on RNA extracted from Day 5 multinucleated osteoclasts (after the lifting and removing of mononuclear cells). Transcript levels of *RANK, NFATc1, DC-STAMP*, and *TRAP* were compared between the three conditions, calculated as the relative difference to *GAPDH* expression, and normalized to Ctrl osteoclasts. RANK is a receptor constitutively expressed in osteoclast progenitors that drives osteoclastogenesis upon binding with RANKL [37,39,40]. Interestingly, the mRNA expression of *RANK* in multinucleated osteoclasts under E2-WD was ~2-fold greater than that in multinucleated osteoclasts with no E2 treatment (Ctrl) (Figure 3A). Furthermore, *RANK* mRNA expression in multinucleated osteoclasts under E2-WD was ~3.5-fold greater than that in cells under continuous E2 treatment (Figure 3A). RANKL binding to RANK activates multiple signaling cascades to induce *NFATc1* expression, the master transcription factor of osteoclastogenesis [41,42,43] that in turn regulates the expression of various osteoclast-specific genes including *DC-STAMP*, a key regulator of osteoclast cell–cell fusion [44,45]. We found no significant difference in the level of *NFATc1* or *DC-STAMP* mRNA expression in multinucleated osteoclasts between the Ctrl, E2, and E2-WD group, although its levels trended higher in osteoclasts under E2 withdrawal (Figure 3B,C). NFATc1 also regulates the expression of TRAP, an enzyme secreted by osteoclasts during bone resorption that is a marker of mature osteoclasts [46,47]. We found a significant increase (~2-fold) in *TRAP* mRNA expression in multinucleated osteoclasts under E2-WD compared to osteoclasts under continuous E2 treatment (Figure 3D). Thus, the presence and withdrawal of E2 had impacts on osteoclast differentiation genes, most notably *RANK* and *TRAP* mRNA expression levels.

Together, these findings showed that continuous E2 treatment lowered the fusion (Figure 1C) and resorption function (Figure 2C,D) of osteoclasts in comparison to osteoclasts with no E2 treatment (Ctrl). These E2-mediated effects were reverted in osteoclasts under E2-WD, which showed increased fusion (Figure 1C), mineral resorption (Figure 2C,D), and osteoclast differentiation markers (Figure 3A,D). Thus, we utilized this E2-WD model for subsequent experiments to examine the mechanism behind enhanced osteoclast activity when E2 is experimentally removed.

### 2.3. E2-WD Shortened Podosome Lifespan and Enhanced Podosome Belt Formation/Stability in Osteoclasts

**Figure 4:**
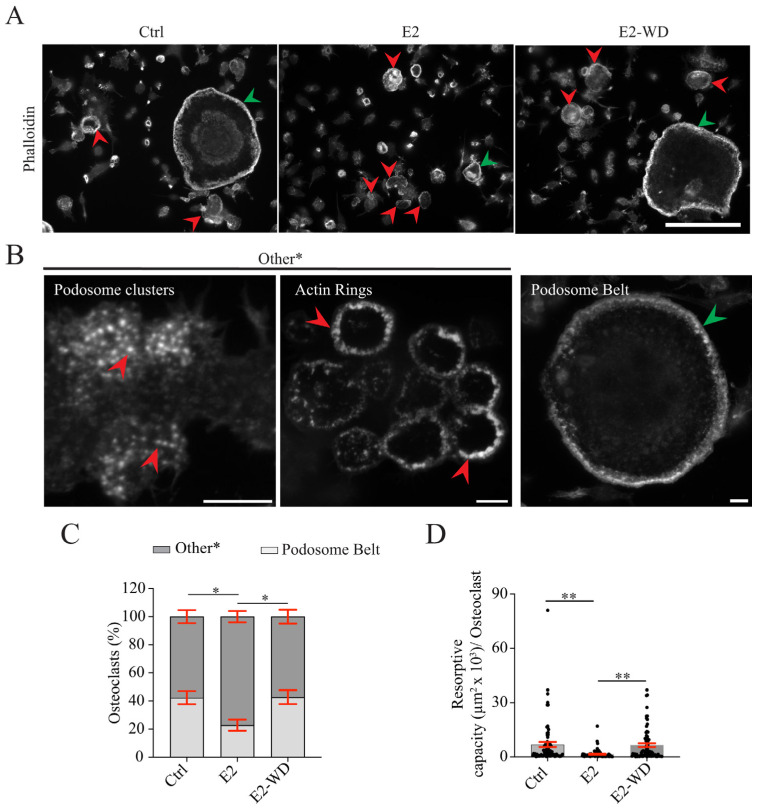
A greater frequency of osteoclasts with a podosome belt and greater resorption capacity in osteoclasts that underwent E2-WD as compared to those continuously treated with E2. (**A**) Representative immunofluorescence images of osteoclasts in the Ctrl, E2, and E2-WD conditions. Cells were fixed after a 4-day differentiation period and stained with phalloidin (F-actin). Green and red arrowheads indicate osteoclasts presenting belt and other podosome organization (ring and/or cluster), respectively. Scale bars = 100 µm. (**B**) Representative immunofluorescence images illustrating the different categories of podosome organization in osteoclasts. Scale bars = 10 µm. (**C**) Stacked bar graph showing the mean frequency of osteoclasts presenting either belt or other podosome organization (ring and/or cluster) in the Ctrl, E2, and E2-WD conditions (*n* ≥ 100 osteoclasts). (**D**) Graph representing the resorptive capacity (µm^2^) (area of podosome belt or the sum of the area of F-actin rings) of osteoclasts under indicated conditions. Data presented are the mean ± SEM for three biological replicates. χ^2^ contingency test (**C**) and the one-way ANOVA test (**D**) were performed and were followed by Tukey’s multiple comparison test (* *p* < 0.05, ** *p* < 0.001).

We next examined the cytoskeletal machinery that promotes osteoclastic bone resorption in the presence, or upon withdrawal, of E2. Resorbing osteoclasts formed stable F-actin structures of a single belt that encompassed the entire cell periphery (Figure 4A) [48,49]. The large belts are termed the “sealing zone” (when osteoclasts are on bone) or the “podosome belt” (when osteoclasts are cultured on glass) due to its enrichment of podosomes [49,50,51]. Podosomes are dynamic F-actin-based structures involved in osteoclast cell adhesion, migration, and bone resorption and they also assemble into less stable structures called clusters and F-actin rings (Figure 4A,B) [48,49]. To examine E2 effects on podosome belt formation/stability, we quantified the proportion of osteoclasts (≥3–8 nuclei) with either a peripheral F-actin-containing podosome belt or “other” (F-actin ring or clusters) podosome structures, identified using phalloidin staining (Figure 4A). We found that there was a significantly smaller proportion of osteoclasts under continuous E2 treatment containing podosomes organized into a belt structure than in the Ctrl condition (Figure 4C). In turn, continuous E2 treatment induced a greater frequency of osteoclasts with podosomes organized in F-actin rings or clusters than that observed in the Ctrl condition (Figure 4C). Interestingly, in osteoclasts that underwent E2-WD, a significantly larger population of osteoclasts contained a podosome belt than that observed in osteoclasts under continuous E2 treatment (Figure 4C). Also, there was a significantly smaller population of osteoclasts with F-actin rings or podosome clusters than that of cells from the E2 group (Figure 4C). The frequency of osteoclasts containing a podosome belt structure or other podosome structure was similar between the Ctrl and E2-WD group (Figure 4C). These findings suggest that continuous E2 treatment suppressed podosome belt formation/stability in osteoclasts and that this effect was reversed under E2-WD to levels resembling fully activated osteoclasts in Ctrl.

Next, we examined the resorptive capacity of osteoclasts (total area of podosome belt or sum of the area of F-actin rings) under the various conditions for cells cultured on glass to test if the presence of these F-actin structures correlated with our mineral resorption assays (Figure 2). Consistently, we found that the resorptive capacity of osteoclasts was significantly reduced under continuous E2 treatment in comparison to the Ctrl group (Figure 4D). Furthermore, the resorptive capacity of osteoclasts under E2-WD was significantly greater than that in the E2 group (Figure 4D), which is in line with the increase in resorptive function observed for the osteoclasts under E2-WD on bone substrate (Figure 2C,D).

### 2.4. Osteoclasts under E2-WD Had Lower Levels of Active RhoA Compared to Those Continuously Exposed to E2

**Figure 5:**
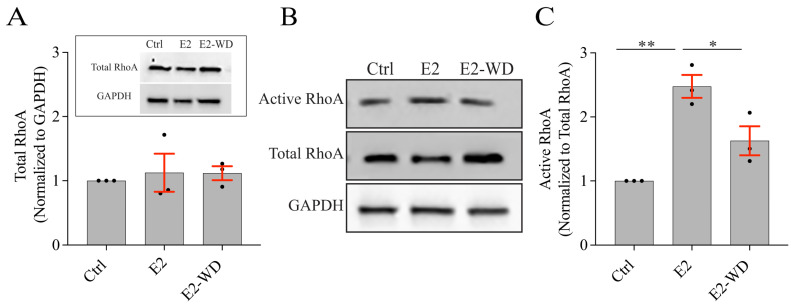
Lower levels of active RhoA in osteoclasts that underwent E2-WD compared to osteoclasts under continuous E2 treatment. (**A**) Representative inverted immunoblots and densitometry analysis of total RhoA normalized with GAPDH using lysates of Ctrl, E2, and E2-WD osteoclasts collected on Day 5. (**B**) Representative inverted immunoblots showing active RhoA, total RhoA, and GAPDH using lysates of Ctrl, E2, and E2-WD osteoclasts collected on Day 5. (**C**) Densitometry analysis of active RhoA normalized with total RhoA. Data presented as fold change ± SEM from three biological replicates. *p*-values were calculated using the one-way ANOVA test, which was followed by Tukey’s multiple comparison test (* *p* < 0.05, ** *p* < 0.001).

We next examined the activity of the Rho GTPase, RhoA, which is known to regulate F-actin organization in osteoclasts [52]. Lower levels of active RhoA (i.e., RhoA-GTP) supports podosome belt formation in osteoclasts on glass surfaces [53,54]. First, we tested whether total RhoA was altered under E2 and E2-WD conditions (Figure 5A). Total RhoA was normalized to the GAPDH (loading control) level, and no significant differences in total RhoA levels were found between the Ctrl, E2, and E2-WD conditions (Figure 5A). suggesting that E2 is not modulating the expression of RhoA. Next, we examined whether active RhoA levels differed between cells in the the E2 condition, which showed a suppression in podosome belt assembly/stability as compared to osteoclasts in the E2-WD condition. To quantify RhoA activation in osteoclasts, we performed a pull-down activation assay for GTP-bound RhoA (i.e., active RhoA) and western blot analysis for RhoA-GTP, total RhoA, and GAPDH (Figure 5B).

Densitometry analysis of triplicate blots and statistical analysis revealed that the levels of RhoA-GTP (normalized to total RhoA levels) in osteoclasts under continuous E2 treatment were significantly greater (~2.5-fold) than those in cells from the Ctrl condition (Figure 5C). Interestingly, the level of RhoA-GTP was significantly reduced in osteoclasts that underwent E2-WD in comparison to the E2 group (Figure 5C). There was no significant difference in the level of RhoA-GTP between the Ctrl and E2-WD conditions (Figure 5C). These results suggest that in osteoclasts under continuous E2 treatment, there is increased levels of activation of RhoA, which is suppressed upon E2-WD.

### 2.5. Effects of Estrogen on Microtubule (MT) Growth and Podosome Lifespan in Osteoclasts

**Figure 6:**
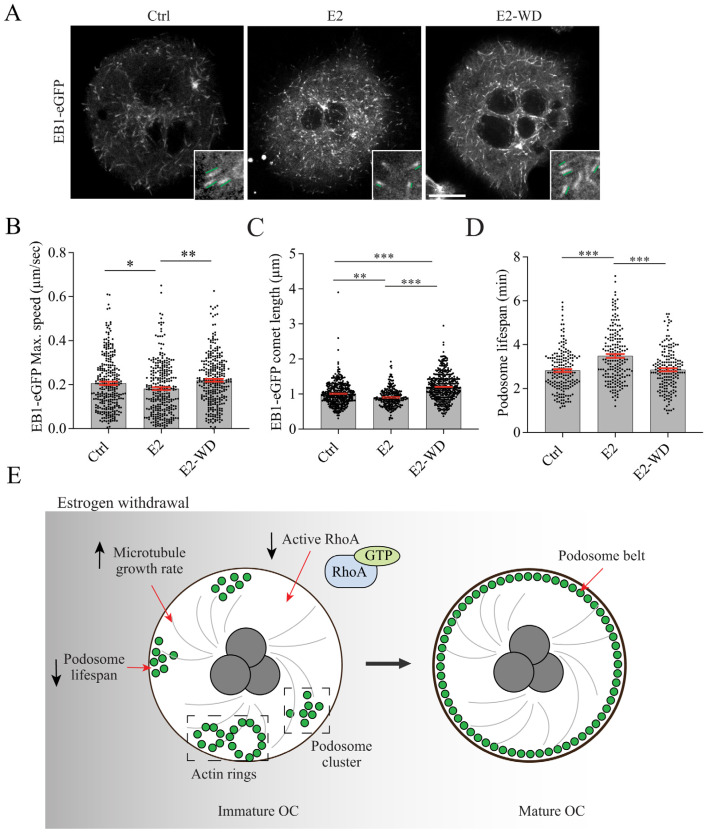
E2-WD altered MT and podosome dynamics in osteoclasts. (**A**) Representative immunofluorescence images of osteoclasts expressing EB1-eGFP under various conditions. Inset: enlarged view of EB1-eGFP comets. Parallel lines indicate the estimated lengths of the EB1-eGFP comets. (**B**) Graph representing maximum speed (µm/s) of EB1-GFP tracked comets in Ctrl, E2, and E2-WD osteoclasts. (**C**) Length of EB1-eGFP comets in osteoclasts under indicated conditions. Each dot represents a MT plus-end comet (*n* ≥ 259 individual comet from ≥14 osteoclasts per condition) from three biological replicates. (**D**) Average lifespan of podosomes in osteoclasts under indicated conditions. Each dot represents a time between appearance and disappearance of LifeAct-RFP-labeled actin podosome (*n* ≥ 180 podosomes from 6–8 osteoclasts per condition) from three biological replicates. Data presented are the mean ± SEM (**B**–**D**). A one-way ANOVA test was performed followed by Tukey’s multiple comparison test (* *p* < 0.05, ** *p* < 0.001, *** *p* < 0.0001). Scale bars = 10 µm. (**E**) Schematic of our findings where the removal of estrogen from cell culture media reduces RhoA activation and increases MT growth rate, which lowers the lifespan of podosomes to enhance podosome belt assembly/stability in osteoclasts.

MT dynamic instability is necessary for podosome formation in adherent macrophages [55] and proper podosome belt and sealing zone organization since treatment of osteoclasts with MT depolymerizing (nocodazole) or stabilizing (taxol) drugs leads to podosome belt disassembly and reorganization into the less resorptive structures: rings or clusters [56,57]. Thus, to analyze MT dynamics in osteoclasts under our E2 conditions, we monitored MT growth in live cells. We transfected osteoclasts with EB1-eGFP on Day 3 of differentiation and conducted live-cell imaging on Day 5 in osteoclasts (≥3–8 nuclei) expressing EB1-eGFP. Plus-end tracking proteins (+TIPS), such as EB1, accumulate at the growing plus ends of MTs and also regulate podosome dynamics [58,59]. EB1-eGFP comets in the osteoclast cytosol showed continuous movements towards the cell periphery indicative of MT polymerization (Figure 6A and Video S1). Using the TrackMate plugin in ImageJ, we measured EB1 growth speed and found that the continuous treatment of osteoclasts with E2 slowed MT polymerization in comparison to osteoclasts in the Ctrl and E2-WD groups (Figure 6B). In osteoclasts under continuous E2 treatment, the maximum EB1 growth speed was significantly lower than that in the Ctrl condition (mean ± SEM of 0.18 ± 0.0068 compared to 0.21 ± 0.0069 µm/s, respectively) (Figure 6B). The EB1 growth speed in osteoclasts that underwent E2-WD was on average 0.22 ± 0.0066 µm/s (mean ± SEM) and was significantly greater than the growth speeds of MTs in osteoclasts from the E2 group (Figure 6B). Strikingly, we noticed unique phenotypes in EB1-eGFP comet length across the Ctrl, E2, or E2-WD conditions. In osteoclasts continuously treated with E2, the EB1-eGFP comets were noticeably shorter than those under E2-withdrawal conditions and control osteoclasts, as shown in Figure 6A,C and Video S1. We also observed that EB1-eGFP comets were significantly longer in osteoclasts that underwent E2-WD as compared to control osteoclasts (Figure 6A,C and Video S1).

Building on the known link between MT dynamics and podosome life span [60], we next examined how E2 presence and withdrawal affected individual podosome dynamics. Podosomes have an average lifespan of 3 min in murine primary osteoclasts [60] and 2.5 min in RAW-derived osteoclasts [61]. Interestingly, as podosomes become incorporated into podosome belts, their individual lifespan is reduced, and they become further destabilized [50,62]. We examined E2 effects on general podosome stability by measuring the lifespan of individual, resolved podosomes within clusters (not associated with an F-actin ring or podosome belt). To measure podosome lifespan, RAW cells stably expressing LifeAct-RFP were differentiated into osteoclasts under the Ctrl, E2, or E2-WD conditions, and live-cell imaging was performed on Day 5 to quantify podosome dynamics. Consistent with the literature [60], the average lifespan of podosomes under the Ctrl condition was 2.82 ± 0.07 min (mean ± SEM) (Figure 6D). In osteoclasts that underwent continuous E2 treatment, the average lifespan of podosomes was 3.49 ± 0.08 min, which was significantly longer than that of the podosomes in the Ctrl group (Figure 6D). The average lifespan of podosomes in osteoclasts that underwent E2-WD was significantly reduced to 2.85 ± 0.07 min (Figure 6D). These findings suggest that E2 stabilizes podosomes through the increase in podosome lifespan, and this effect is lost in osteoclasts under E2-WD.

## 3. Discussion

In this work, we developed an acute cell culture system to study the cellular mechanisms driving the estrogen-mediated suppression of osteoclasts and their activity upon estrogen withdrawal. Previous studies examined estrogen withdrawal impacts on osteoclasts used primary bone-marrow-derived macrophages (BMDMs) from ovariectomized mice [23,63]. However, primary cells are difficult to extract and have shorter lifespans [64,65]. Furthermore, primary cell-derived osteoclasts are challenging to transfect, hampering in-depth mechanistic analysis [66]. In contrast, the commercially available immortalized cell line RAW 264.7 cells are easy to culture and generate large populations of bone-resorbing osteoclasts in a short span of time [33,67]. Within our minimal and robust 5-day model, continuous E2 treatment of RAW cell-derived osteoclasts led to a significant decrease in osteoclast fusion and bone mimetic resorption, consistent with previous research studying the effects of estrogen on osteoclasts [18,20,21,23,68]. Using a 2-day exposure to physiological E2 levels followed by a 2-day E2 removal led to higher osteoclast formation and mineral resorption than osteoclasts that underwent continuous E2 treatment, mirroring observed deregulated/hyperactive osteoclast function in osteoclasts under estrogen-deficient conditions [20,68,69,70]. Gene expression analysis revealed that the osteoclast differentiation markers were highly expressed after estrogen withdrawal, in some cases even higher than that in control cells. While all differentiation markers measured showed the same trends, some did not reach statistical significance in differences, which warrants future protein analysis in osteoclasts under the influence of estrogen. Taken together, these data give us confidence that this is a useful system for the rapid and deep mechanistic analysis of osteoclast activation upon estrogen withdrawal.

Using live-cell imaging analysis, we observed a shorter podosome lifespan in osteoclasts experiencing E2-WD as compared to cells continuously exposed to E2. Others have shown that as podosomes associate with a podosome belt, their lifespan is reduced, and they are further destabilized [60,71,72,73]. These data support our observations and suggest that E2-WD increases the dynamicity of podosomes, allowing spatial reorganization from clustered podosomes and rings into a peripheral F-actin-rich podosome belt during osteoclast maturation, which correlates with their enhanced resorptive capacity [74]. Consistent with previous reports, we found podosome organization and turnover to coincide with changes in MT dynamics and GTP-RhoA levels, which we further speculate together or independently drive podosome turnover in osteoclasts under E2-WD.

There are conflicting reports on the effects of estrogen on RhoA activation. E2 was reported to increase RhoA activation in mouse hippocampal cells and human umbilical vein endothelial cells [75,76,77]. In osteoclasts, RhoA activity is associated with marked changes in cytoskeletal organization and expression of constitutively active Rho-promoted podosome assembly and bone resorption [78], while inhibiting Rho activity was reported to impair the formation of podosome ring structures and reduce osteoclast resorptive activity [52,78,79].

Despite the inhibitory effect of E2 on podosome organization and consistent with decreased osteoclast resorption activity, E2 appears to stimulate elevated levels of RhoA-GTP. We found greater levels of active RhoA in osteoclasts under continuous E2 treatment than in osteoclasts that underwent E2-WD and fully activated control osteoclasts. Our results agree with observations in osteoclasts in which the inhibition of Rho activity enhanced podosome belt formation [53,54]. This observation suggests the existence of potential additional mechanisms that locally regulate Rho GTPase activation near the podosome or other pathways that diminish RhoA activity towards downstream targets. E2 binding to ERα in human endothelial cells triggers ERα to bind with and activate the G-protein, Gα_13_, which in turn activates RhoA [75]. Interestingly, although Gα_13_ is highly expressed in murine BMDM-derived osteoclasts, its activity suppresses RhoA activation in these cells [80]. GPER1 has also been shown to modulate RhoA activity [81,82]. The activation of GPER1 with the agonists, tamoxifen and G1 in human hepatic stellate cells and human foreskin fibroblasts, respectively, suppresses RhoA activation [81,82]. Future identification of the estrogen receptor(s) and RhoA GEFs stimulated in our system will help resolve the mechanism driving E2-mediated RhoA activation.

We further provide a link between E2 and MT dynamics and podosome turnover in osteoclasts. Tracking MTs in live cells showed that continuous E2 exposure slowed MT growth speed and altered the distribution of EB1-eGFP comets at the growing MT tip as compared to osteoclasts under E2-WD. E2 is known to impact MT dynamics directly, and it was found that in vitro, purified tubulin treated with 10 nM of E2, similar to the concentration used in our model, reduced MT polymerization [83]. Furthermore, exposing human osteosarcoma cells to a E2 derivative (formed by the dimerization of E2) was shown to result in reduced MT dynamicity, with the speed of MT growth being slowed [84], similar to what we observed in the EB1-eGFP comets in the continuously E2-treated osteoclasts. Comet length of EB1 or its homologue EB3 was reported to positively correlate with the MT growth rate [85,86,87]. Consistent with our result of shorter EB1-eGFP comets and slower MT growth speed under E2 conditions, we speculate that E2 exposure would influence the recruitment of other MT +TIPs proteins that depend on EB1 to localize to the MT plus end such as CLIP-170 [87,88] and would alter MT interactions with the cell cortex [89,90].

MTs are positive regulators of podosome formation and stability. Inhibiting MT growth and dynamics with low doses of nocodazole were reported to inhibit podosome belt assembly in osteoclasts [58] and multinucleated giant cells [91]. Furthermore, in human peripheral-blood-monocyte-derived macrophages, the contact of MT plus ends with podosomes was found to induce podosome dissolution [92]. At the molecular level, EB1 is critical for MT-dependent regulation of podosome dynamics, as it interacts and forms a complex with the podosome proteins cortactin and vinculin in osteoclasts, and disrupting MT dynamic instability disrupts complex formation [58]. Binding of the MT motor KIF1C to non-muscle myosin II is also important for podosome dynamics [92]. Others have shown that faster MT growth speeds are correlated with increased podosome lifespans in osteoclasts generated from cortactin knockout mice [58]. More in line with what we observed, deletion of the β-tubulin isotype, TUBB6, in RAW-264.7-cell-derived osteoclasts was found to cause reduced a MT growth speed, which was associated with an increase in podosome lifespan [61].

Based on our findings, we propose a model in which continuous E2 treatments inhibit podosome belt assembly/stability, with this being reversed in osteoclasts undergoing E2-WD. E2-WD influences RhoA activation and MT growth, which impact podosome stability, leading to enhanced podosome belt formation and bone resorption in osteoclasts (Figure 6E). Further mechanistic analysis of this in vitro cell-culture, post-menopause model will enhance our understanding of osteoclast activation and allow rapid and affordable testing for therapeutic interventions for bone-wasting disorders.

## 4. Materials and Methods

### 4.1. Reagents and Antibodies

Dulbecco’s Modified Eagle Medium (DMEM) and fetal bovine serum (FBS) were purchased from Wisent Inc. (St-Bruno, QC, Canada). Phosphate-buffered saline (PBS), Alpha Modified Eagle Medium (AMEM), Minimum Essential Medium (MEM) (without L-glutamine and phenol red) and TrypLE™ Select Enzyme (1×, no phenol red) were purchased from ThermoFisher Scientific (Waltham, MA, USA). Recombinant receptor activator of nuclear factor kappa-B ligand (RANKL) was produced from BL21 *Escherichia coli* transformed with a pGEX-GST-hRANKL vector (a gift from Morris Manolson, Faculty of Dentistry, University of Toronto). 17β-estradiol was purchased from MilliporeSigma Canada Co. (Oakville, ON, Canada). Alexa Fluor^®^ 488 phalloidin was purchased from ThermoFisher Scientific. 4′,6-Diamidine-2′-phenylindole dihydrochloride (DAPI) was purchased from MilliporeSigma. DAKO Fluorescence mounting media was purchased from Agilent Technologies Canada Inc. (Mississauga, ON, Canada). RhoA Activation Assay Kit (Bead pull-down format) was purchased from Cytoskeleton, Inc. (Denver, CO, USA). Goat anti-mouse horseradish peroxidase (HRP) was purchased from Jackson ImmunoResearch Laboratories Inc. (West Grove, PA, USA), and anti-GAPDH antibody loading control was purchased from Abcam (Cambridge, UK). For cell transfection, FuGENE^®^ HD reagent was purchased from Promega Corporation (Fitchburg, WI, USA). The plasmid expressing human EB1-GFP (JB131) was a gift from Tim Mitchison and Jennifer Tirnauer (Addgene plasmid # 39299).

### 4.2. RAW 264.7 Cell Culture and Osteoclast Differentiation

The RAW 264.7 (RAW) murine macrophage cell line, obtained from American Type Culture Collection (ATCC, Manassas, VA, USA), was cultured in complete DMEM containing 10% heat-inactivated FBS and maintained in 5% CO_2_ and 37 °C conditions. To generate osteoclasts, confluent RAW cells were cultured into 12-well tissue culture plates at an average cell density of 3 × 10^4^ cells/well in AMEM supplemented with 10% FBS. Cells were stimulated on Day 1 at the time of plating with 25 ng/mL of RANKL and cultured for 4 days, with media and RANKL replenishment on Day 3. The control group (Ctrl; untreated osteoclasts) was prepared as previously stated. The continuous E2 treatment group (E2) was prepared in the same manner as that of Ctrl; however, 10 nM of 17β-estradiol, a concentration within the physiological range in mouse serum [38], was added each day. Cells in the E2-WD group, were treated with 10 nM of 17β-estradiol for 2 days, followed by washes in AMEM to remove residual traces before cells were grown for additional 2 days in the absence of 17β-estradiol. The experiment was terminated, and cells were fixed on Day 5.

### 4.3. Immunofluorescence and Fixed Cell Imaging

Cells differentiated on coverslips were fixed in 4% paraformaldehyde (PFA) in PBS and permeabilized with 0.1% Triton X-100 and 100 mM glycine for 20 min. After fixation and subsequent washes in 1x PBS, cells were blocked with 5% FBS in PBS for an hour. Cells were incubated with Alexa Fluor^®^ 488 phalloidin (1:500) in 1% FBS in PBS to label F-actin. Cells were washed with PBS and with double-distilled water (ddH_2_O). Nuclei were stained with DAPI (1:1000), diluted in ddH_2_O, washed with ddH_2_O, and then mounted onto coverslips using DAKO. Epifluorescence images were taken with a 40× 1.4 NA immersion lens using a Zeiss inverted AxioObserver Z1 microscope (Carl Zeiss Canada, Toronto, ON, Canada) with an Axiocam 506 mono-camera and configured using Zeiss Zen 3.1 software for image capture. Image visualization and analysis were performed using Fiji/ImageJ version 2.9.0 and 2.14.0 (National Institutes of Health, Bethesda, MD, USA).

Fusion index (%) was calculated by dividing the number of nuclei in osteoclasts (defined to contain ≥ 3 nuclei) by the total number of nuclei in the field of view. Actin organization was categorized into a podosome belt, actin rings, and clusters and scored based on F-actin staining in fixed osteoclasts. Osteoclasts scored as containing a podosome belt if showed a continuous row of podosomes at the cell periphery. Osteoclasts with “other” podosome structures contained podosomes organized into smaller rings and/or clusters. The resorptive capacity of osteoclasts was measured in F-actin-stained, fixed osteoclasts. Using the Freehand selections tool in ImageJ, the perimeter of the podosome belt was manually traced, and the area of the podosome belt was measured and recorded as its resorptive capacity. For osteoclasts containing multiple actin rings, the area of individual actin rings was measured as above and the sum of the area of F-actin rings was recorded as the osteoclasts’ resorptive capacity.

### 4.4. Biomimetic Calcium Phosphate Substrate, Osteoclast Culture, and Resorption Pit Analysis

To quantify osteoclast resorption activity in vitro, a biomimetic substrate for the inorganic mineral component of bone, hydroxyapatite, was prepared by coating glass coverslips with a thin layer of calcium phosphate (CaP). The procedure for coating coverslips was carried out as previously described [93,94]. Briefly, a 2.5 × concentrated simulated body fluid (SBF) solution was prepared by mixing Tris buffer solution (50 mM), calcium stock solution (25 mM of CaCl_2_∙2H_2_O, 1.37 M of NaCl, 15 mM of MgCl_2_∙6H_2_O in Tris buffer), and phosphate stock solution (11.1 mM of Na_2_HPO_4_∙2H_2_O, 42 mM of NaHCO_3_ in Tris buffer) in a 2:1:1 ratio. Freshly prepared 2.5 × SBF solution was added to 12-well plates at room temperature for 2 days, with daily replenishment. A thin layer of CaP formed, which acted as a nucleation layer for the final calcium phosphate solution (CPS, 2.25 of mM Na_2_HPO_4_∙2H_2_O, 4 mM of CaCl_2_∙2H_2_O, 0.14 M of NaCl, and 50 mM of Tris in MilliQ water; a pH of 7.4). CPS solution was added for 2 days at room temperature. Then, the CaP-coated coverslips were sterilized by adding 70% ethanol for 20 min, washed twice with distilled water, and dried inside a 37 °C unhumidified incubator overnight. Prior to plating, CaP-coated coverslips were equilibrated with AMEM supplemented with 50% FBS overnight at 37 °C.

The starting density of RAW cells was optimized to achieve an osteoclast differentiation time frame similar to what was observed on glass coverslips. RAW cells were plated at a cell density of 6 × 10^4^ cells/well in AMEM supplemented with 10% FBS and were simulated with 50 ng/mL RANKL on Day 1, with media and RANKL replenishment on Day 3. Cells under E2 and E2-WD conditions were given the same estrogen treatment regime as mentioned in the earlier cell culture section. On day 5, cells were removed using 2% hypochlorite and then treated with 2.5% (*w*/*v*) silver nitrate for 20 min to observe resorption pits. Coverslips were imaged using a Zeiss AxioObserver Z1 inverted epifluorescence microscope with DIC at 20× magnification. The number and area of resorption pits were analyzed using ImageJ.

### 4.5. Quantitative RT-PCR

RAW cells were plated on 10 cm dishes at a density of 3 × 10^4^ cells/mL, differentiated and treated with E2 using similar treatments and timeline as described earlier. To examine alterations in expression of genes in mature, multinucleated osteoclasts, on Day 5, 1 mL of TrypLE Select (Gibco, ThermoFisher Scientific) was added to remove the less adherent mononuclear cells before RNA extraction was performed using the PureLink RNA Kit (Invitrogen, ThermoFisher Scientific). The purity and quantity of RNA were determined with a DeNovix DS-11FX NanoDrop spectrophotometer/fluorometer (DeNovix Inc., Wilmington, DE, USA) prior to cDNA synthesis. Subsequently, 1 µg of total RNA was used to generate cDNA using the SuperScript III First-Strand Synthesis SuperMix for quantitative RT-PCR (qRT-PCR) (Invitrogen, ThermoFisher Scientific) using the primer sets outlined in Table 1.

qRT-PCR reaction was carried out using PowerUp™ SYBR Green Master Mix (Applied Biosystems, ThermoFisher Scientific) in the QuantStudio 3 thermal cycler (ThermoFisher Scientific) with an initial denaturing temperature of 95 °C for 2 min followed by 40 cycles of 15 s at 95 °C, 15 s at 55 °C, and 20 s at 60 °C. Melting curve analysis was completed with each qRT-PCR run to ensure the absence of nonspecific primer binding. The gene expression profile was visualized and analyzed using the Relative Quantification analysis module on Thermo Fisher Cloud. *GAPDH* (Real Time Primers, LLC, Melrose Park, PA, USA) was used as the reference gene. The expressions of genes of interest were presented as fold changes using the comparative Ct method (2^−ΔΔCt^). Three independent biological replicates were performed for each experiment, and each reaction was run in triplicate.

### 4.6. RhoA Activation Assay

GTP-bound active RhoA was quantified using the RhoA Pull-Down Activation Assay Kit according to the manufacturer’s protocol. Briefly, 3 × 10^4^ RAW cells/mL were plated on 10 cm dishes using similar treatments and timeline as described earlier. On Day 5, cells were placed on ice, washed once with PBS, and lysed using 50 mM of Tris pH 7.5, 10 mM of MgCl_2_, 0.5 M of NaCl, and 2% IGEPAL. Lysate was clarified by centrifugation at 10,000× *g* for 1 min at 4 °C, supernatant collected, and subsequently, the concentration was determined using the *DC* protein assay (Bio-Rad Laboratories, Inc., Hercules, CA, USA). About 800 µg of lysates was incubated with Rhotekin-RBD protein beads for 1 h at 4 °C before rhotekin-RBD beads were pelleted by centrifugation at 5000× *g* at 4 °C for 1 min, washed once with wash buffer (25 mM of Tris pH 7.5, 30 mM of MgCl_2_, 40 mM of NaCl) and pelleted again at 5000× *g* at 4 °C for 3 min. The supernatant was removed, and the beads were resuspended in 2× Laemmli buffer (BioRad Laboratories Inc., Hercules, CA, USA) and boiled for 2 min.

Samples were separated on 4–20% Mini-PROTEAN^®^ TGX™ (BioRad) and transferred to nitrocellulose membranes. Nitrocellulose membranes were then blocked for 1 h in Tris-buffered saline with 0.1% Tween^®^ 20 Detergent (TBST) containing 5% BSA and probed with mouse monoclonal RhoA (1:500) and mouse monoclonal GAPDH (1:1000) overnight at 4 °C, washed, and incubated with the corresponding HRP-conjugated secondary antibodies (1:10,000) for 1 h. Chemiluminescence signals were imaged using a ChemiDoc Imaging System (Bio-Rad). Densitometry analysis of protein bands from three biological replicates were measured using ImageJ.

### 4.7. EB1-GFP Transfection, Live Cell Imaging, and Analysis

On Day 3, differentiating RAW cells plated on 35 mm glass-bottom plates were chemically transfected with a 1.1 µg/dish of EB1-GFP using FuGENE^®^ HD (Promega, WI, USA). On Day 5, an hour prior to imaging, the medium was replaced with phenol red-free medium. Osteoclasts were imaged using the Quorum WaveFX-X1 spinning disc confocal system (Quorum Technologies Inc., Guelph, ON, Canada) with a 63× 1.4 NA oil immersion lens in 5% CO_2_ and 37 °C conditions. Z-stack time lapse images of osteoclasts (≥3–8 nuclei) expressing EB1-GFP were acquired at 4 sec intervals.

Analysis of EB1-eGFP were performed in cells with comparable expression levels. Time lapse images were processed in ImageJ via the tracking of EB1-eGFP comets using the TrackMate plug-in. Comets with an estimated diameter of 1 µm and appearing in at least two consecutive frames were included. Data on total distance (in µm), maximum velocity (µm/s), mean velocity (µm/s), and forward progression of 30 comets/osteoclast were recorded. Using maximum intensity projections of Z-stack confocal images of live osteoclasts, the line tool in ImageJ was used to manually trace the length of EB1-eGFP comets. The length of EB1-eGFP was measured for at least 259 comets that were chosen randomly in 14–21 osteoclasts from three independent experiments. EB1-eGFP comets were measured in cells with a low background signal, and only comets that were in focus for at least two consecutive frames were considered for length analysis.

### 4.8. Measuring Podosome Lifespan

To visualize the F-actin structures in osteoclasts, LifeAct-RFP-stably-transfected RAW macrophages were plated in 35 mm glass-bottom dishes and treated using similar regimens and timelines as described above. One hour before live imaging, the medium was replaced with phenol red-free medium. Osteoclasts were imaged using the Quorum WaveFX-X1 spinning disc confocal system with a 63× 1.4 NA oil immersion lens in 5% CO_2_ and in 37 °C conditions. Time lapse images of osteoclasts (≥3–10 nuclei) expressing LifeAct-RFP were acquired at 4 s intervals for 10 min before analysis using ImageJ. The lifespan of individual podosomes (not part of an actin ring or podosome belt) was measured by the manual tracking of the interval between the appearance and disappearance of the individual podosomes. The lifespan of 20–30 podosomes per osteoclast was measured, and 6–8 different osteoclasts were included per condition.

### 4.9. Statistical Analysis

For statistical analysis, in the comparison of three groups or more, a non-parametric one-way ANOVA test was used and followed by Tukey’s test for multiple comparisons. The mean ± standard error of the mean (SEM) was plotted. Prism (version 7.03, GraphPad Software Inc., La Jolla, CA, USA) was used to conduct the statistical analyses, with *p* < 0.05 being considered statistically significant.

## Figures and Tables

**Table 1:** Osteoclast-specific and control qPCR primer sequences (5′-3′).

*DC-STAMP*	F-TACGTGGAGAGAAGCAAGGAA	R-ACACTGAGACGTGGTTTAGGAAT
*NFATc1*	F-CCCGTCACATTCTGGTCCAT	R-CAAGTAACCGTGTAGCTGCACAA
*RANK*	F-CACAGACAAATGCAAACCTTG	R-GTGTTCTGGAACCATCTTCCTCC
*TRAP*	F-ACGGCTACTTGCGGTTTCA	R-TCCTTGGGAGGCTGGTCTT
*GAPDH*	F-AATGAGCCTTCCTCTGCTCT	R-AACTGGCTATTCAGCTGTGG

## Data Availability

All data generated or analyzed during this study are available to share, and a detailed protocol can be granted through request to the author.

## Supplementary Materials

The following supporting information can be downloaded at https://www.mdpi.com/article/10.3390/ijms25116134/s1.

## References

[1] Florencio-Silva R., Sasso G.R.D.S., Sasso-Cerri E., Simões M.J., Cerri P.S. (2015). Biology of Bone Tissue: Structure, Function, and Factors That Influence Bone Cells. BioMed Res. Int..

[2] Kenkre J.S., Bassett J.H.D. (2018). The bone remodelling cycle. Ann. Clin. Biochem..

[3] Salari N., Ghasemi H., Mohammadi L., Behzadi M.H., Rabieenia E., Shohaimi S., Mohammadi M. (2021). The global prevalence of osteoporosis in the world: A comprehensive systematic review and meta-analysis. J. Orthop. Surg. Res..

[4] Raisz L.G. (2005). Science in medicine Pathogenesis of osteoporosis: Concepts, conflicts, and prospects. J. Clin. Investig..

[5] Garnero P., Sornay-Rendu E., Chapuy M.C., Delmas P.D. (1996). Increased bone turnover in late postmenopausal women is a major determinant of osteoporosis. J. Bone Miner. Res..

[6] Cui J., Shen Y., Li R. (2013). Estrogen synthesis and signaling pathways during aging: From periphery to brain. Trends Mol. Med..

[7] Simpson E., Rubin G., Clyne C., Robertson K., O’Donnell L., Jones M., Davis S. (2000). The Role of Local Estrogen Biosynthesis in Males and Females. Trends Endocrinol. Metab..

[8] Purohit A., Flanagan A.M., Reed M.J. (1992). Estrogen synthesis by osteoblast cell lines. Endocrinology.

[9] Janssen J.M., Bland R., Hewison M., Coughtrie M.W., Sharp S., Arts J., Pols H.A., van Leeuwen J.P. (1999). Estradiol formation by human osteoblasts via multiple pathways: Relation with osteoblast function. J. Cell. Biochem..

[10] Eyre L.J., Bland R., Bujalska I.J., Sheppard M.C., Stewart P.M., Hewison M. (1998). Characterization of aromatase and 17β-hydroxysteroid dehydrogenase expression in rat osteoblastic cells. J. Bone Miner. Res..

[11] Fuentes N., Silveyra P. (2019). Estrogen receptor signaling mechanisms. Advances in Protein Chemistry and Structural Biology.

[12] Björnström L., Sjöberg M. (2005). Mechanisms of Estrogen Receptor Signaling: Convergence of Genomic and Nongenomic Actions on Target Genes. Mol. Endocrinol..

[13] Safe S., Kim K. (2008). Non-Classical Genomic Estrogen Receptor (ER)/Specificity Protein and ER/Activating Protein-1 Signaling Pathways. J. Mol. Endocrinol..

[14] Parry T., Ledee D., Willis M.S., Portman M.A. (2017). Nuclear Receptors and the Adaptive Response of the Heart. Endocrinology of the Heart in Health and Disease: Integrated, Cellular, and Molecular Endocrinology of the Heart.

[15] Feng Y., Gregor P. (1997). Cloning of a Novel Member of the G Protein-Coupled Receptor Family Related to Peptide Receptors 1. Biochem. Biophys. Res. Commun..

[16] Filardo E.J., Quinn J.A., Bland K.I., Frackelton A.R., Williams R. (2000). Estrogen-Induced Activation of Erk-1 and Erk-2 Requires the G Protein-Coupled Receptor Homolog, GPR30, and Occurs via Trans-Activation of the Epidermal Growth Factor Receptor through Release of HB-EGF. Mol. Endo-Crinol..

[17] Weitzmann M.N., Pacifici R. (2006). Estrogen deficiency and bone loss: An inflammatory tale. J. Clin. Investig..

[18] Nakamura T., Imai Y., Matsumoto T., Sato S., Takeuchi K., Igarashi K., Harada Y., Azuma Y., Krust A., Yamamoto Y. (2007). Estrogen Prevents Bone Loss via Estrogen Receptor α and Induction of Fas Ligand in Osteoclasts. Cell.

[19] Martin-Millan M., Almeida M., Ambrogini E., Han L., Zhao H., Weinstein R.S., Jilka R.L., O’Brien C.A., Manolagas S.C. (2010). The estrogen receptor-α in osteoclasts mediates the protective effects of estrogens on cancellous but not cortical bone. Mol. Endocrinol..

[20] Kim H.N., Ponte F., Nookaew I., Ucer Ozgurel S., Marques-Carvalho A., Iyer S., Warren A., Aykin-Burns N., Krager K., Sardao V.A. (2020). Estrogens decrease osteoclast number by attenuating mitochondria oxidative phosphorylation and ATP production in early osteoclast precursors. Sci. Rep..

[21] Kameda T., Mano H., Yuasa T., Mori Y., Miyazawa K., Shiokawa M., Nakamaru Y., Hiroi E., Hiura K., Kameda A. (1997). Estrogen inhibits bone resorption by directly inducing apoptosis of the bone-resorbing osteoclasts. J. Exp. Med..

[22] Hughes D.E., Dai A., Tiffee J.C., Li H.H., Mundy G.R., Boyce B.F. (1996). Estrogen promotes apoptosis of murine osteoclasts mediated by TGF–β. Nat. Med..

[23] Shevde N.K., Bendixen A.C., Dienger K.M., Pike J.W. (2000). Estrogens suppress RANK ligand-induced osteoclast differentiation via a stromal cell independent mechanism involving c-Jun repression. Proc. Natl. Acad. Sci. USA.

[24] Srivastava S., Toraldo G., Weitzmann M.N., Cenci S., Ross F.P., Pacifici R. (2001). Estrogen Decreases Osteoclast Formation by Down-regulating Receptor Activator of NF-κB Ligand (RANKL)-induced JNK Activation. J. Biol. Chem..

[25] Palacios V.G., Robinson L.J., Borysenko C.W., Lehmann T., Kalla S.E., Blair H.C. (2005). Negative regulation of RANKL-induced osteoclastic differentiation in RAW264.7 cells by estrogen and phytoestrogens. J. Biol. Chem..

[26] Saintier D., Khanine V., Uzan B., Ea H.K., de Vernejoul M.C., Cohen-Solal M.E. (2006). Estradiol inhibits adhesion and promotes apoptosis in murine osteoclasts in vitro. J. Steroid Biochem. Mol. Biol..

[27] Gavali S., Gupta M.K., Daswani B., Wani M.R., Sirdeshmukh R., Khatkhatay M.I. (2019). LYN, a key mediator in estrogen-dependent suppression of osteoclast differentiation, survival, and function. Biochim. Et Biophys. Acta-Mol. Basis Dis..

[28] Park H.J., Gholam-Zadeh M., Yoon S.Y., Suh J.H., Choi H.S. (2021). Estrogen decreases cytoskeletal organization by forming an erα/shp2/c-src complex in osteoclasts to protect against ovariectomy-induced bone loss in mice. Antioxidants.

[29] Streicher C., Heyny A., Andrukhova O., Haigl B., Slavic S., Schüler C., Kollmann K., Kantner I., Sexl V., Kleiter M. (2017). Estrogen Regulates Bone Turnover by Targeting RANKL Expression in Bone Lining Cells. Sci. Rep..

[30] Li J.Y., Tawfeek H., Bedi B., Yang X., Adams J., Gao K.Y., Zayzafoon M., Weitzmann M.N., Pacifici R. (2011). Ovariectomy disregulates osteoblast and osteoclast formation through the T-cell receptor CD40 ligand. Proc. Natl. Acad. Sci. USA.

[31] Geoghegan I.P., McNamara L.M., Hoey D.A. (2021). Estrogen withdrawal alters cytoskeletal and primary ciliary dynamics resulting in increased Hedgehog and osteoclastogenic paracrine signalling in osteocytes. Sci. Rep..

[32] Brennan M., Haugh M., O’Brien F., McNamara L. (2014). Estrogen Withdrawal from Osteoblasts and Osteocytes Causes Increased Mineralization and Apoptosis. Horm. Metab. Res..

[33] Hsu H., Lacey D.L., Dunstan C.R., Solovyev I., Colombero A., Timms E., Tan H.L., Elliott G., Kelley M.J., Sarosi I. (1999). Tumor necrosis factor receptor family member RANK mediates osteoclast differentiation and activation induced by osteoprotegerin ligand. Proc. Natl. Acad. Sci. USA.

[34] Geoghegan I.P., Hoey D.A., McNamara L.M. (2019). Estrogen deficiency impairs integrin αvβ3-mediated mechanosensation by osteocytes and alters osteoclastogenic paracrine signalling. Sci. Rep..

[35] Fiorino C., Harrison R.E. (2016). E-cadherin is important for cell differentiation during osteoclastogenesis. Bone.

[36] Jeganathan S., Fiorino C., Naik U., Sun H.S., Harrison R.E. (2014). Modulation of osteoclastogenesis with macrophage M1- and M2-inducing stimuli. PLoS ONE.

[37] Vincent C., Kogawa M., Findlay D.M., Atkins G.J. (2009). The generation of osteoclasts from RAW 264.7 precursors in defined, serum-free conditions. J. Bone Miner. Metab..

[38] Haisenleder D.J., Schoenfelder A.H., Marcinko E.S., Geddis L.M., Marshall J.C. (2011). Estimation of estradiol in mouse serum samples: Evaluation of commercial estradiol immunoassays. Endocrinology.

[39] Boyce B.F., Xing L. (2008). Functions of RANKL/RANK/OPG in bone modeling and remodeling. Arch. Biochem. Biophys..

[40] Dougall W.C., Glaccum M., Charrier K., Rohrbach K., Brasel K., De Smedt T., Daro E., Smith J., Tometsko M.E., Maliszewski C.R. (1999). RANK is essential for osteoclast and lymph node development. Genes Dev..

[41] Jones D.H., Kong Y.-Y., Penninger J.M. (2002). Role of RANKL and RANK in bone loss and arthritis. Ann. Rheum. Dis..

[42] Zhao C., Gao H., Liu Y., Papoutsi Z., Jaffrey S., Gustafsson J.-Å., Dahlman-Wright K. (2010). Genome-Wide Mapping of Estrogen Receptor-β–Binding Regions Reveals Extensive Cross-Talk with Transcription Factor Activator Protein-1. Cancer Res..

[43] Kim J.H., Kim N. (2016). Signaling Pathways in Osteoclast Differentiation. Chonnam Med. J..

[44] Mensah K.A., Ritchlin C.T., Schwarz E.M. (2010). RANKL induces heterogeneous DC-STAMP ^lo^ and DC-STAMP ^hi^ osteoclast precursors of which the DC-STAMP ^lo^ precursors are the master fusogens. J. Cell. Physiol..

[45] Yagi M., Miyamoto T., Sawatani Y., Iwamoto K., Hosogane N., Fujita N., Morita K., Ninomiya K., Suzuki T., Miyamoto K. (2005). DC-STAMP is essential for cell–cell fusion in osteoclasts and foreign body giant cells. J. Exp. Med..

[46] Matsuo K., Galson D.L., Zhao C., Peng L., Laplace C., Wang K.Z.Q., Bachler M.A., Amano H., Aburatani H., Ishikawa H. (2004). Nuclear Factor of Activated T-Cells (NFAT) Rescues Osteoclastogenesis in Precursors Lacking c-Fos. J. Biol. Chem..

[47] Hayman A. (2008). Tartrate-Resistant Acid Phosphatase (TRAP) and the Osteoclast/Immune Cell Dichotomy. Autoimmunity.

[48] Song R., Gu J., Liu X., Zhu J., Wang Q., Gao Q., Zhang J., Cheng L., Tong X., Qi X. (2014). Inhibition of osteoclast bone resorption activity through osteoprotegerin-induced damage of the sealing zone. Int. J. Mol. Med..

[49] Takito J., Inoue S., Nakamura M. (2018). The Sealing Zone in Osteoclasts: A Self-Organized Structure on the Bone. Int. J. Mol. Sci..

[50] Georgess D., Machuca-Gayet I., Blangy A., Jurdic P. (2014). Podosome organization drives osteoclast-mediated bone resorption. Cell Adhes. Migr..

[51] Schachtner H., Calaminus S.D.J., Thomas S.G., Machesky L.M. (2013). Podosomes in adhesion, migration, mechanosensing and matrix remodeling. Cytoskeleton.

[52] Wang J., Xu C., Zhang J., Bao Y., Tang Y., Lv X., Ma B., Wu X., Mao G. (2023). RhoA promotes osteoclastogenesis and regulates bone remodeling through mTOR-NFATc1 signaling. Mol. Med..

[53] Destaing O., Saltel F., Gilquin B., Chabadel A., Khochbin S., Ory S., Jurdic P. (2005). A novel Rho-mDia2-HDAC6 pathway controls podosome patterning through microtubule acetylation in osteoclasts. J. Cell Sci..

[54] Gil-Henn H., Destaing O., Sims N.A., Aoki K., Alles N., Neff L., Sanjay A., Bruzzaniti A., De Camilli P., Baron R. (2007). Defective microtubule-dependent podosome organization in osteoclasts leads to increased bone density in *Pyk2−/−* mice. J. Cell Biol..

[55] Linder S., Hufner K., Wintergerst U., Aepfelbacher M. (2000). Microtubule-dependent formation of podosomal adhesion structures in primary human macrophages. J. Cell Sci..

[56] Batsir S., Geiger B., Kam Z. (2017). Dynamics of the sealing zone in cultured osteoclasts. Cytoskeleton.

[57] Ti Y., Zhou L., Wang R., Zhao J. (2015). Inhibition of Microtubule Dynamics Affects Podosome Belt Formation during Osteoclast Induction. Cell Biochem. Biophys..

[58] Biosse Duplan M., Zalli D., Stephens S., Zenger S., Neff L., Oelkers J.M., Lai F.P.L., Horne W., Rottner K., Baron R. (2014). Microtubule Dynamic Instability Controls Podosome Patterning in Osteoclasts through EB1, Cortactin, and Src. Mol. Cell. Biol..

[59] Vitre B., Coquelle F.M., Heichette C., Garnier C., Chrétien D., Arnal I. (2008). EB1 regulates microtubule dynamics and tubulin sheet closure in vitro. Nat. Cell Biol..

[60] Destaing O., Saltel F., Géminard J.C., Jurdic P., Bard F. (2003). Podosomes display actin turnover and dynamic self-organization in osteoclasts expressing actin-green fluorescent protein. Mol. Biol. Cell.

[61] Maurin J., Morel A., Guérit D., Cau J., Urbach S., Blangy A., Bompard G. (2021). The Beta-Tubulin Isotype TUBB6 Controls Microtubule and Actin Dynamics in Osteoclasts. Front. Cell Dev. Biol..

[62] Luxenburg C., Addadi L., Geiger B. (2006). The molecular dynamics of osteoclast adhesions. Eur. J. Cell Biol..

[63] Chen F., Ouyang Y., Ye T., Ni B., Chen A. (2014). Estrogen inhibits RANKL-induced osteoclastic differentiation by increasing the expression of TRPV5 channel. J. Cell. Biochem..

[64] Chevalier C., Çolakoğlu M., Brun J., Thouverey C., Bonnet N., Ferrari S., Trajkovski M. (2021). Primary mouse osteoblast and osteoclast culturing and analysis. STAR Protoc..

[65] Quan J., Hou Y., Long W., Ye S., Wang Z. (2018). Characterization of different osteoclast phenotypes in the progression of bone invasion by oral squamous cell carcinoma. Oncol. Rep..

[66] Cuetara B.L.V., Crotti T.N., O’Donoghue A.J., McHugh K.P. (2006). Cloning and characterization of osteoclast precursors from the RAW264.7 cell line. Vitr. Cell. Dev. Biol.-Anim..

[67] Collin-Osdoby P., Osdoby P. (2012). RANKL-mediated osteoclast formation from murine RAW 264.7 cells. Methods Mol. Biol..

[68] Piao H., Chu X., Lv W., Zhao Y. (2017). Involvement of receptor-interacting protein 140 in estrogen-mediated osteoclasts differentiation, apoptosis, and bone resorption. J. Physiol. Sci..

[69] Miyamoto H., Suzuki T., Miyauchi Y., Iwasaki R., Kobayashi T., Sato Y., Miyamoto K., Hoshi H., Hashimoto K., Yoshida S. (2012). Osteoclast stimulatory transmembrane protein and dendritic cell-specific transmembrane protein cooperatively modulate cell-cell fusion to form osteoclasts and foreign body giant cells. J. Bone Miner. Res..

[70] Liu L., Zhou L., Yang X., Liu Q., Yang L., Zheng C., Zhao Y., Zhang Z., Luo X. (2018). 17β-estradiol attenuates ovariectomy-induced bone deterioration through the suppression of the ephA2/ephrinA2 signaling pathway. Mol. Med. Rep..

[71] Destaing O., Sanjay A., Itzstein C., Horne W.C., Toomre D., De Camilli P., Baron R. (2008). The tyrosine kinase activity of c-Src regulates actin dynamics and organization of podosomes in osteoclasts. Mol. Biol. Cell.

[72] Luxenburg C., Parsons J.T., Addadi L., Geiger B. (2006). Involvement of the Src-cortactin pathway in podosome formation and turnover during polarization of cultured osteoclasts. J. Cell Sci..

[73] Granot-Attas S., Luxenburg C., Finkelshtein E., Elson A. (2009). Protein tyrosine phosphatase epsilon regulates integrin-mediated podosome stability in osteoclasts by activating Src. Mol. Biol. Cell.

[74] Jurdic P., Saltel F., Chabadel A., Destaing O. (2006). Podosome and sealing zone: Specificity of the osteoclast model. Eur. J. Cell Biol..

[75] Simoncini T., Scorticati C., Mannella P., Fadiel A., Giretti M.S., Fu X.D., Baldacci C., Garibaldi S., Caruso A., Fornari L. (2006). Estrogen receptor α interacts with Gα13 to drive actin remodeling and endothelial cell migration via the RhoA/Rho kinase/moesin pathway. Mol. Endocrinol..

[76] Kramár E.A., Chen L.Y., Brandon N.J., Rex C.S., Liu F., Gall C.M., Lynch G. (2009). Cytoskeletal changes underlie estrogen’s acute effects on synaptic transmission and plasticity. J. Neurosci..

[77] Oviedo P.J., Sobrino A., Laguna-Fernandez A., Novella S., Tarín J.J., García-Pérez M.A., Sanchís J., Cano A., Hermenegildo C. (2011). Estradiol induces endothelial cell migration and proliferation through estrogen receptor-enhanced RhoA/ROCK pathway. Mol. Cell. Endocrinol..

[78] Chellaiah M.A., Soga N., Swanson S., McAllister S., Alvarez U., Wang D., Dowdy S.F., Hruska K.A. (2000). Rho-A Is Critical for Osteoclast Podosome Organization, Motility, and Bone Resorption. J. Biol. Chem..

[79] Zhang D., Udagawa N., Nakamura I., Murakami H., Saito S., Yamasaki K., Shibasaki Y., Morii N., Narumiya S., Takahashi N. (1995). The small gtp-binding protein, RHO p21, is involved in bone resorption by regulating cytoskeletal organization in osteoclasts. J. Cell Sci..

[80] Nakano S., Inoue K., Xu C., Deng Z., Syrovatkina V., Vitone G., Zhao L., Huang X.Y., Zhao B. (2019). G-protein Gα 13 functions as a cytoskeletal and mitochondrial regulator to restrain osteoclast function. Sci. Rep..

[81] Lachowski D., Cortes E., Matellan C., Rice A., Lee D.A., Thorpe S.D., del Río Hernández A.E. (2020). G Protein-Coupled Estrogen Receptor Regulates Actin Cytoskeleton Dynamics to Impair Cell Polarization. Front. Cell Dev. Biol..

[82] Cortes E., Lachowski D., Rice A., Thorpe S.D., Robinson B., Yeldag G., Lee D.A., Ghemtio L., Rombouts K., del Río Hernández A.E. (2019). Tamoxifen mechanically deactivates hepatic stellate cells via the G protein-coupled estrogen receptor. Oncogene.

[83] Kipp J.L., Ramirez V.D. (2003). Estradiol and testosterone have opposite effects on microtubule polymerization. Neuroendocrinology.

[84] Jurášek M., Černohorská M., Řehulka J., Spiwok V., Sulimenko T., Dráberová E., Darmostuk M., Gurská S., Frydrych I., Buriánová R. (2018). Estradiol dimer inhibits tubulin polymerization and microtubule dynamics. J. Steroid Biochem. Mol. Biol..

[85] Urazbaev A., Serikbaeva A., Tvorogova A., Dusenbayev A., Kauanova S., Vorobjev I. (2021). On the Relationship Between EB-3 Profiles and Microtubules Growth in Cultured Cells. Front. Mol. Biosci..

[86] Fong K.W., Au F.K.C., Jia Y., Yang S., Zhou L., Qi R.Z. (2017). Microtubule plus-end tracking of end-binding protein 1 (EB1) is regulated by CDK5 regulatory subunit-associated protein 2. J. Biol. Chem..

[87] Bieling P., Laan L., Schek H., Munteanu E.L., Sandblad L., Dogterom M., Brunner D., Surrey T. (2007). Reconstitution of a microtubule plus-end tracking system in vitro. Nature.

[88] Dixit R., Barnett B., Lazarus J.E., Tokito M., Goldman Y.E., Holzbaur E.L.F. (2009). Microtubule plus-end tracking by CLIP-170 requires EB1. Proc. Natl. Acad. Sci. USA.

[89] Galjart N., Perez F. (2003). A plus-end raft to control microtubule dynamics and function. Curr. Opin. Cell Biol..

[90] Gundersen G.G., Gomes E.R., Wen Y. (2004). Cortical control of microtubule stability and polarization. Curr. Opin. Cell Biol..

[91] Ory S., Destaing O., Jurdic P. (2002). Microtubule dynamics differentially regulates Rho and Rac activity and triggers Rho-independent stress fiber formation in macrophage polykaryons. Eur. J. Cell Biol..

[92] Kopp P., Lammers R., Aepfelbacher M., Woehlke G., Rudel T., Machuy N., Steffen W., Linder S. (2006). The kinesin KIF1C and microtubule plus ends regulate podosome dynamics in macrophages. Mol. Biol. Cell.

[93] Maria S.M., Prukner C., Sheikh Z., Mueller F., Barralet J.E., Komarova S.V. (2014). Reproducible quantification of osteoclastic activity: Characterization of a biomimetic calcium phosphate assay. J. Biomed. Mater. Res.-Part B Appl. Biomater..

[94] Patntirapong S., Habibovic P., Hauschka P.V. (2009). Effects of soluble cobalt and cobalt incorporated into calcium phosphate layers on osteoclast differentiation and activation. Biomaterials.

